# Human immunodeficiency virus infection and risks of morbidity and death in adults with incident heart failure

**DOI:** 10.1093/ehjopen/oeab040

**Published:** 2021-12-01

**Authors:** Harshith R Avula, Andrew P Ambrosy, Michael J Silverberg, Kristi Reynolds, William J Towner, Rulin C Hechter, Michael Horberg, Suma Vupputuri, Thomas K Leong, Wendy A Leyden, Teresa N Harrison, Keane K Lee, Sue Hee Sung, Alan S Go

**Affiliations:** 1 Department of Cardiology, Kaiser Permanente Walnut Creek Medical Center, Walnut Creek, CA 94596, USA; 2 Department of Cardiology, Kaiser Permanente San Francisco Medical Center, San Francisco, CA 94115, USA; 3 Division of Research, Kaiser Permanente Northern California, 2000 Broadway, Oakland, CA 94612, USA; 4 Department of Research and Evaluation, Kaiser Permanente Southern California, Pasadena, CA 91101, USA; 5 Department of Infectious Disease, Kaiser Permanente Los Angeles Medical Center, Los Angeles, CA 90027, USA; 6 Department of Clinical Science, Kaiser Permanente Bernard J. Tyson School of Medicine, Pasadena, CA 91101, USA; 7 Department of Health Systems Science, Kaiser Permanente Bernard J. Tyson School of Medicine, Pasadena, CA 91101, USA; 8 Mid-Atlantic Permanente Research Institute, Kaiser Permanente Mid-Atlantic States, Rockville, MD 20852, USA; 9 Department of Cardiology, Kaiser Permanente Santa Clara Medical Center, Santa Clara, CA 95051, USA; 10 Department of Epidemiology, Biostatistics and Medicine, University of California, San Francisco, San Francisco, CA 94158, USA; 11 Department of Medicine, Stanford University, Palo Alto, CA 94304, USA

**Keywords:** Human immunodeficiency virus, Heart failure, Mortality, Hospitalization, Emergency department visit, Epidemiology

## Abstract

**Aims:**

Human immunodeficiency virus (HIV) increases the risk of heart failure (HF), but whether it influences subsequent morbidity and mortality remains unclear.

**Methods and results:**

We investigated the risks of hospitalization for HF, HF-related emergency department (ED) visits, and all-cause death in an observational cohort of incident HF patients with and without HIV using data from three large US integrated healthcare delivery systems. We estimated incidence rates and adjusted hazard ratios (aHRs) by HIV status at the time of HF diagnosis for subsequent outcomes. We identified 448 persons living with HIV (PLWH) and 3429 without HIV who developed HF from a frequency-matched source cohort of 38 868 PLWH and 386 586 without HIV. Mean age was 59.5 ± 11.3 years with 9.8% women and 31.8% Black, 13.1% Hispanic, and 2.2% Asian/Pacific Islander. Compared with persons without HIV, PLWH had similar adjusted rates of HF hospitalization [aHR 1.01, 95% confidence interval (CI): 0.81–1.26] and of HF-related ED visits [aHR 1.22 (95% CI: 0.99–1.50)], but higher adjusted rates of all-cause death [aHR 1.31 (95% CI: 1.08–1.58)]. Adjusted rates of HF-related morbidity and all-cause death were directionally consistent across a wide range of CD4 counts but most pronounced in the subset with a baseline CD4 count <200 or 200–499 cells/μL.

**Conclusion:**

In a large, diverse cohort of adults with incident HF receiving care within integrated healthcare delivery systems, PLWH were at an independently higher risk of all-cause death but not HF hospitalizations or HF-related ED visits. Future studies investigating modifiable HIV-specific risk factors may facilitate more personalized care to optimize outcomes for PLWH and HF.

## Graphical Abstract

**Graphical Abstract:**
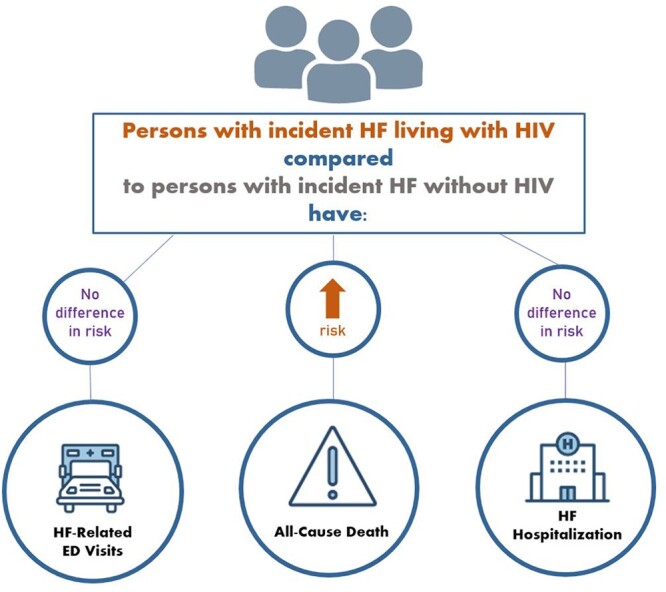

## Introduction

Advances in antiretroviral therapy (ART) have favourably altered the course of human immunodeficiency virus (HIV) infection and survival among persons living with HIV (PLWH),^1–4^ who are increasingly at risk of developing other disabling chronic conditions, including heart failure (HF).^5–8^ Recent studies support that the increased risk of incident HF among PLWH is not entirely explained by greater burden of ischaemic heart disease alone.^6^^,^^7^^,^^9^ Reasons for the excess risk of HF in PLWH are not fully understood but may be due, at least in part, to the effects of chronic immune activation on myocardial function and fibrosis,^10^^,^^11^ myocyte invasion by HIV and aberrations in cellular signalling pathways,^12^^,^^13^ or ART-related cardiotoxicity.^14^^,^^15^

Importantly, even less is known about whether HIV infection independently affects outcomes after developing HF. Studies of women and predominantly Black or Hispanic adults hospitalized for HF have reported higher risks of recurrent HF hospitalization, cardiovascular death, and death in PLWH.^16^^,^^17^ Short-term risk of adverse HF outcomes among PLWH after hospitalization for HF has also been reported to be associated with an individual’s level of immunodeficiency or ART regimen.^17^^,^^18^ These findings, however, are not generalizable to the contemporary incident HF population among PLWH and have been largely derived from single-centre investigations of only hospitalized adults with HF, with relatively short follow-up and limited patient diversity.

Among patients with incident HF within a contemporary, ethnically diverse parent cohort study comparing PLWH with matched adults without HIV, we addressed these knowledge gaps by evaluating the influence of HIV status on long-term risks of subsequent HF hospitalization, HF-related emergency department (ED) visits, and all-cause death.

## Materials and methods

### Study overview

The HIV HEART Study is a National Heart, Lung and Blood Institute (NHLBI)-funded observational study (R01 HL132640) conducted at three Kaiser Permanente (KP) integrated healthcare delivery systems that provide comprehensive care tracked through their electronic medical record (EMR) systems. Kaiser Permanente Northern California serves >4.5 million members at 21 medical centres and >260 medical offices; KP Southern California serves >4.6 million members at 15 medical centres and >230 medical offices; and KP Mid-Atlantic States serves 800 000 members at 36 medical offices. The HIV HEART study was designed to address evidence gaps on the incidence, prevention, and treatment of HF in PLWH. Specifically, the objectives are to characterize the incidence of HF among PLWH and patients without HIV, to identify HIV-specific risk factors for incident HF including long-term immunodeficiency and the composition and adherence to ART, and to evaluate rates of HF hospitalizations, HF-related ED visits, and death among PLWH and persons without HIV who develop incident HF. The purpose of the present analysis is to compare outcomes among PLWH and persons without HIV after developing HF.

The study was approved by institutional review boards of participating institutions, and waiver of informed consent was obtained because of the nature of the study. The data underlying this article are available in the article.

### Identification of persons living with human immunodeficiency virus and persons without human immunodeficiency virus

The KP Virtual Data Warehouse (VDW) served as a primary data source for subject identification and characterization. The VDW is comprised of site-specific EMR-based datasets populated with linked demographic, administrative, outpatient pharmacy, outpatient laboratory test results, and healthcare utilization data for KP members.^19^ Each KP site has also implemented comprehensive HIV registries that maintain up-to-date lists of all PLWH, HIV transmission risk factors, dates of known HIV infection, acquired immunodeficiency syndrome diagnoses, HIV-related lab and pharmacy data, and associated EMR data. Human immunodeficiency virus registries include all known members with HIV since the early 1980s for KP Northern California, since 1998 in KP Mid-Atlantic States, and since 2000 for KP Southern California, with confirmation by manual chart review or provider review of case lists. To study patients treated in the current ART era, we included all eligible adult (≥21 years) PLWH between 2000 and 2016.

To create a comparable baseline population without HIV from which incident HF cases were identified, adult members without HIV from the same KP source populations and study period were frequency-matched up to 10:1 to PLWH based on calendar year (i.e. year of start of follow-up for adults with HIV), age (±1 year), gender, race, and primary KP treating facility to account for possible practice differences across sites.

### Follow-up, outcomes, and covariates

Using a previously validated approach,^20^^,^^21^ we identified newly diagnosed HF based on being hospitalized with a primary discharge diagnosis of HF or having ≥3 outpatient visits coded for HF with at least one visit with a cardiologist using pre-specified *International Classification of Disease (ICD)-9/10* codes (codes available on request). We previously demonstrated a positive predictive value ≥95% of HF for hospitalizations with a primary discharge diagnosis of HF.^22^ Index date was defined as the date of the first HF diagnosis between 2000 and 2016 and all patients with pre-existing HF at index date were excluded from the matched cohorts prior to analysis. Follow-up occurred from index date through 31 December 2016, with censoring at 10 years of accrued follow-up, health plan disenrolment, death, or the end of administrative study follow-up.

Available index and time-updated information on demographic characteristics, comorbidity, laboratory data, medication use, and vital signs or specific therapies received were obtained using *ICD*-*9/10* and *Current Procedural Terminology* codes and EMR data using validated algorithms.^21^^,^^23–25^ Data on left ventricular ejection fraction (LVEF), if available, were ascertained from results of echocardiograms, radionuclide scintigraphy, other nuclear imaging modalities, and left ventriculography. We categorized patients as HF with preserved ejection fraction, HF with mildly reduced ejection fraction, or HF with reduced ejection fraction consistent with current guidelines.^26^

All cases of incident HF were followed for the development of outcomes of HF hospitalization (i.e. primary discharge diagnosis), HF-related ED visits (i.e. primary or secondary discharge diagnosis), and all-cause death. Hospitalizations and ED visits were comprehensively identified from the VDW using previously described diagnostic codes. Deaths were systematically captured from EMR, administrative, Social Security Administration vital status, and state death certificates.^25^^,^^27^

### Statistical approach

All analyses were conducted using SAS, version 9.4 (Cary, NC, USA). We compared baseline characteristics between PLWH and adults without HIV using *t*-tests or Wilcoxon rank-sum tests for continuous variables and χ^2^ tests for ordinal variables.

We next calculated annualized incidence rates (per 100 person-years) of each outcome with associated 95% confidence intervals (CIs) (i.e. including all observed events), and also compared Kaplan–Meier curves of survival free of each outcome between groups using a log-rank test (i.e. time-to-first event analysis). To evaluate the independent association of HIV infection with adverse HF outcomes and potential explanatory factors, we performed extended Cox regression models that adjusted for the following baseline and time-updated variables, as appropriate: KP site and calendar era, demographic characteristics (age, gender, and self-reported race), neighbourhood-level socioeconomic status (income level and educational attainment), lifestyle factors (smoking status, alcohol abuse, and illicit drug use), baseline medical history (i.e. myocardial infarction, unstable angina, coronary artery bypass surgery, percutaneous coronary intervention, atrial fibrillation/flutter, valvular heart disease, peripheral artery disease, ischaemic stroke or transient ischaemic attack, diabetes mellitus, hypertension, dyslipidaemia, depression, dementia, chronic liver disease, chronic lung disease, hypothyroidism, hyperthyroidism, hospitalization for bleeding, cancer, and receipt of cardiac implantable electronic devices), outpatient vital signs (systolic blood pressure and body mass index), kidney function (estimated glomerular filtration rate and proteinuria), LVEF category, and receipt of targeted medications (i.e. angiotensin-converting enzyme inhibitors/angiotensin II receptor blockers, beta-blockers, calcium channel blockers, diuretics, alpha antagonists, aldosterone receptor blockers, statins, non-statin lipid-lowering agents, anticoagulants, antiplatelet agents, diabetes medications and non-steroidal anti-inflammatory drugs).

Given the potential influence of baseline levels of immunosuppression and HIV disease severity on HF outcomes and death, we repeated analyses with PLWH categorized based on the most recent CD4 cell count obtained within the 6 months before the index HF diagnosis date.^28^^,^^29^

## Results

### Cohort assembly and baseline characteristics

**Figure 1:**
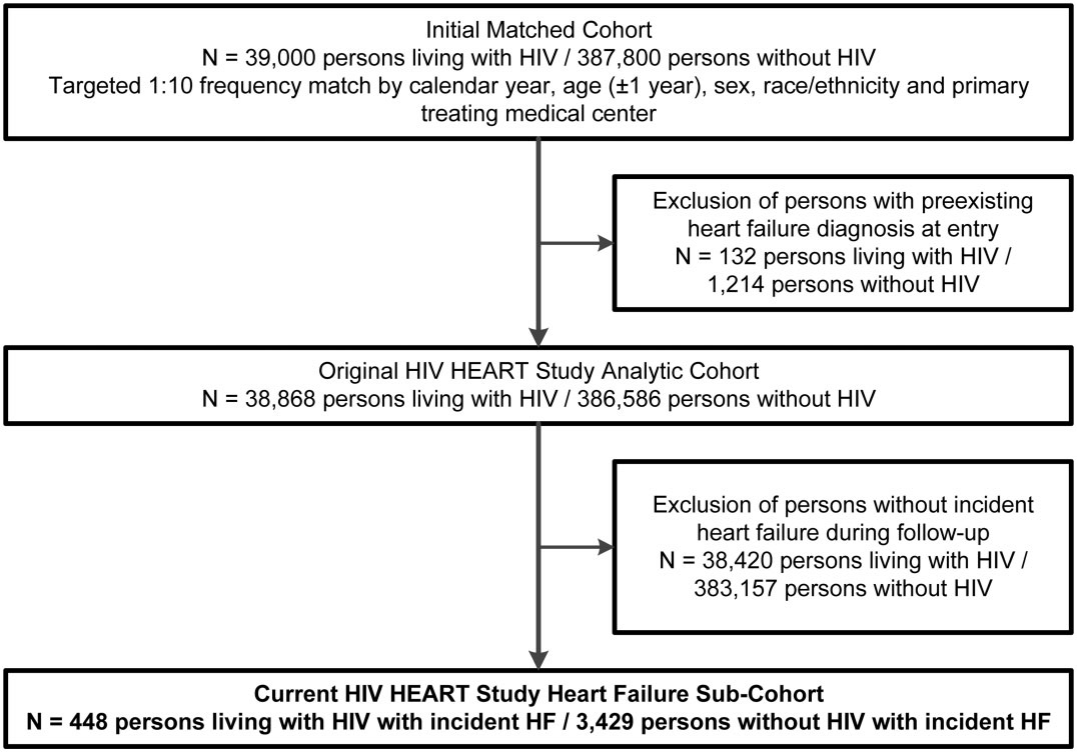
Cohort assembly of persons living with HIV and persons without HIV who were diagnosed with incident heart failure.

From an initial study population of 38 868 PLWH and 386 586 matched persons without HIV, we identified 3877 incident HF cases (448 PLWH, 3429 persons without HIV) (*Figure 1*). Median duration of follow-up after being diagnosed with HF was 3.1 years [interquartile range (IQR): 1.2–6.6 years], mean age was 59.5 ± 11.3 years, and there was broad racial/ethnic diversity (47.0% White, 31.8% Black, 13.1% Hispanic, and 2.2% Asian/Pacific Islander) (*Table 1*). Among these incident HF patients, compared with persons without HIV, PLWH with HF tended to be younger, were more likely to be female, and were more likely to have non-cardiac comorbidities including chronic liver disease, chronic lung disease, depression, and a history of cancer. Persons living with HIV were also more likely to have a history of illicit drug use and had a lower median household income than persons without HIV. Finally, PLWH were less likely than persons without HIV to have clinical cardiovascular risk factors including documented dyslipidaemia, hypertension, and diabetes mellitus. Information on LVEF was unavailable in 40.2% of HF patients but did not differ between PLWH or persons without HIV (*P* = 0.20). Among PLWH, >75% were receiving ART and the median CD4 count was 401 (IQR: 208–616 cells/μL) at index date.

**Table 1:** Characteristics of persons with incident heart failure who are living with or without HIV

	Overall (*N* = 3877)	Adults with incident heart failure and HIV (*N* = 448)	Adults with incident heart failure and no HIV (*N* = 3429)	*P*-value
Mean (SD) age, years	59.5 (11.3)	57.3 (11.6)	59.8 (11.2)	<0.001
Sex				
Female	381 (9.8)	65 (14.5)	316 (9.2)	<0.001
Male	3496 (90.2)	383 (85.5)	3113 (90.8)	
Race/ethnicity				
White	1824 (47.0)	217 (48.4)	1607 (46.9)	<0.001
Black	1233 (31.8)	127 (28.3)	1106 (32.3)	
Hispanic	506 (13.1)	47 (10.5)	459 (13.4)	
Asian/Pacific Islander	84 (2.2)	13 (2.9)	71 (2.1)	
Other/unknown	230 (5.9)	44 (9.8)	186 (5.4)	
Low educational attainment				
No	2582 (66.6)	288 (64.3)	2294 (66.9)	<0.05
Yes	821 (21.2)	88 (19.6)	733 (21.4)	
Missing	474 (12.2)	72 (16.1)	402 (11.7)	
Low median household income				
No	2996 (77.3)	307 (68.5)	2689 (78.4)	<0.001
Yes	407 (10.5)	69 (15.4)	338 (9.9)	
Missing	474 (12.2)	72 (16.1)	402 (11.7)	
Left ventricular ejection fraction				
Preserved (>50%)	1013 (26.1)	109 (24.3)	904 (26.4)	0.20
Mildly reduced (41–49%)	346 (8.9)	45 (10.0)	301 (8.8)	
Reduced (<40%)	959 (24.7)	98 (21.9)	861 (25.1)	
Unknown	1559 (40.2)	196 (43.8)	1363 (39.7)	
CD4 count, cells/μL				
Median (IQR)	Not Applicable	401 (208–616)	Not Applicable	
<50	Not Applicable	19 (4.2)	Not Applicable	
50–199	Not Applicable	81 (18.1)	Not Applicable	
200–499	Not Applicable	162 (36.2)	Not Applicable	
≥500	Not Applicable	154 (34.4)	Not Applicable	
Unknown	Not Applicable	32 (7.1)	Not Applicable	
HIV RNA copies, n/mL				
Median (IQR)	Not Applicable	74 (47–2098)	Not Applicable	
<75	Not Applicable	267 (59.6)	Not Applicable	
75–199	Not Applicable	18 (4.0)	Not Applicable	
200–499	Not Applicable	12 (2.7)	Not Applicable	
≥500	Not Applicable	117 (26.1)	Not Applicable	
Unknown	Not Applicable	34 (7.6)	Not Applicable	
Medical history				
Acute myocardial infarction	548 (14.1)	71 (15.8)	477 (13.9)	0.27
Coronary artery by pass graft surgery	199 (5.1)	15 (3.3)	184 (5.4)	0.07
Percutaneous coronary intervention	438 (11.3)	52 (11.6)	386 (11.3)	0.83
Atrial fibrillation or flutter	840 (21.7)	60 (13.4)	780 (22.7)	<0.001
Stroke/transient ischaemic attack	202 (5.2)	20 (4.5)	182 (5.3)	0.45
Peripheral artery disease	204 (5.3)	22 (4.9)	182 (5.3)	0.72
Hypertension	2856 (73.7)	287 (64.1)	2569 (74.9)	<0.001
Dyslipidaemia	2596 (67.0)	278 (62.1)	2318 (67.6)	<0.05
Diabetes mellitus	1602 (41.3)	129 (28.8)	1473 (43.0)	<0.001
Chronic lung disease	1061 (27.4)	151 (33.7)	910 (26.5)	<0.01
Chronic liver disease	301 (7.8)	85 (19.0)	216 (6.3)	<0.001
Diagnosed dementia	90 (2.3)	20 (4.5)	70 (2.0)	<0.01
Diagnosed depression	626 (16.1)	137 (30.6)	489 (14.3)	<0.001
Tobacco use				
Ever smoker	1584 (40.9)	187 (41.7)	1397 (40.7)	0.45
Non-smoker	1143 (29.5)	121 (27.0)	1022 (29.8)	
Missing	1150 (29.7)	140 (31.3)	1010 (29.5)	
Documented substance use				
Illicit drug use	394 (10.2)	88 (19.6)	306 (8.9)	<0.001
Alcohol abuse	589 (15.2)	61 (13.6)	528 (15.4)	0.32
Baseline therapy for HIV				
No HIV medication	Not Applicable	110 (24.6)	Not Applicable	
Non-Protease inhibitor regimen	Not Applicable	169 (37.7)	Not Applicable	
Protease inhibitor regimen	Not Applicable	169 (37.7)	Not Applicable	
Baseline medication use				
ACE inhibitor	1693 (43.7)	181 (40.4)	1512 (44.1)	0.14
Angiotensin II receptor blocker	425 (11.0)	39 (8.7)	386 (11.3)	0.10
Beta blocker	2002 (51.6)	212 (47.3)	1790 (52.2)	0.05
Calcium channel blocker	981 (25.3)	99 (22.1)	882 (25.7)	0.10
Diuretic	1715 (44.2)	177 (39.5)	1538 (44.9)	<0.05
Aldosterone receptor blocker	137 (3.5)	20 (4.5)	117 (3.4)	0.26
Statin	1776 (45.8)	176 (39.3)	1600 (46.7)	<0.01
Diabetic therapy	1161 (29.9)	80 (17.9)	1081 (31.5)	<0.001
Systolic blood pressure, mmHg				
Mean (SD)	129.4 (21.6)	125.5 (20.9)	129.8 (21.6)	<0.01
Missing	1329 (34.3)	172 (38.4)	1157 (33.7)	
Body mass index, kg/m^2^				
Mean (SD)	31.5 (8.3)	27.1 (6.8)	32.0 (8.3)	<0.001
Missing	1398 (36.1)	181 (40.4)	1217 (35.5)	
Estimated glomerular filtration rate, mL/min/ 1.73 m^2^				
Mean (SD)	72.9 (25.4)	73.7 (25.2)	72.8 (25.4)	0.56

Values are no. (%) unless otherwise indicated.

HIV, human immunodeficiency virus; IQR, interquartile range; SD, standard deviation.

### Human immunodeficiency virus status and heart failure outcomes

**Figure 2:**
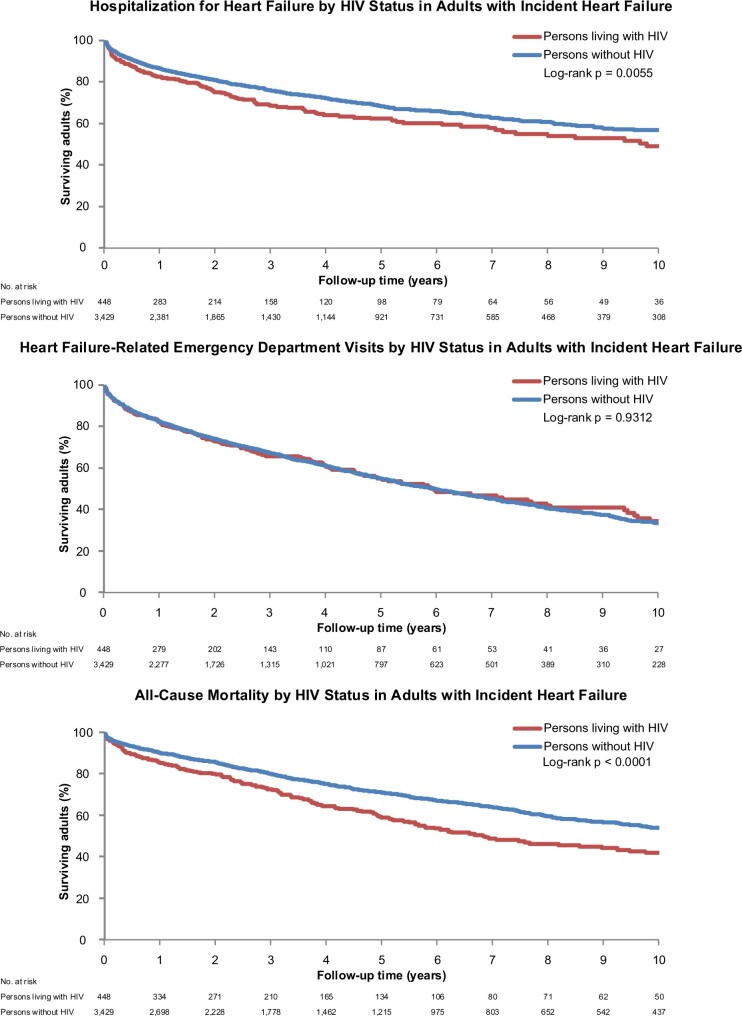
Kaplan–Meier curves for hospitalization for heart failure, heart failure-related emergency department visits, and all-cause death by HIV status after developing incident heart failure.

*Figure 2* shows unadjusted Kaplan–Meier curves for time to first HF hospitalization, HF-related ED visit, and all-cause death among patients with incident HF based on HIV status.

**Figure 3:**
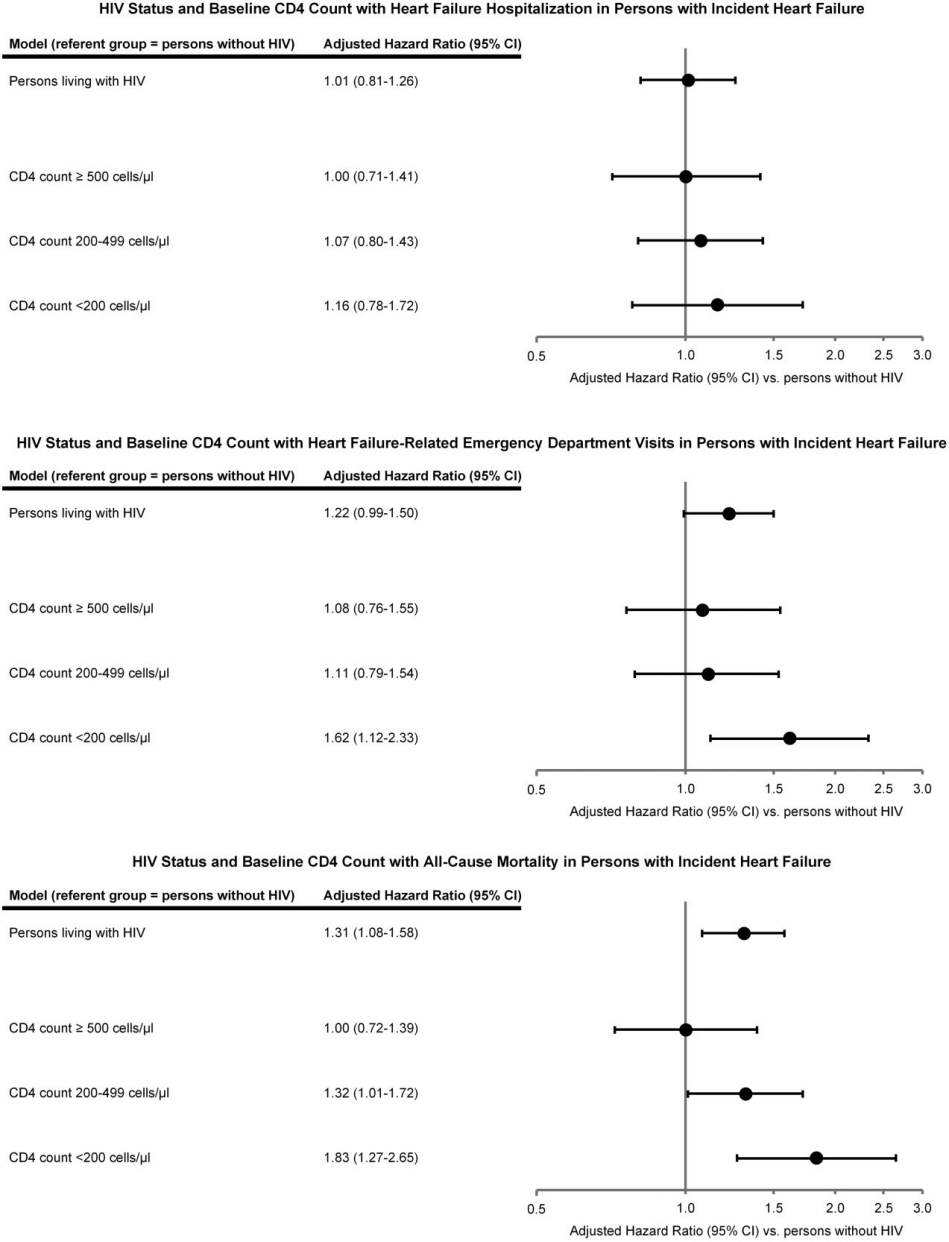
Association between HIV status, baseline CD4 count, and clinical outcomes in adults with incident heart failure. Persons living with HIV with incident heart failure were at increased risk of heart failure-related emergency department visits and all-cause death but not heart failure hospitalizations compared with persons without HIV. These findings were directionally consistent across a wide range of baseline CD4 counts but may be more pronounced among the subset of patients with the highest levels of immunosuppression.

The unadjusted rate (per 100 person-years) of HF hospitalizations was not significantly different among PLWH [14.2 (95% CI: 12.5–16.1)] compared with persons without HIV [13.2 (95% CI: 12.6–13.8), *P* = 0.14], which was confirmed in multivariable analysis [adjusted hazard ratio (aHR) 1.01 (95% CI: 0.81–1.26), *P* = 0.94] (*Figure 3*).

The unadjusted rate (per 100 person-years) of HF-related ED visits was significantly higher among PLWH compared with persons without HIV [33.5 (95% CI: 30.9–36.42) vs. 24.6 (95% CI: 23.8–25.4), respectively, *P* < 0.001]. In contrast, the adjusted risk of HF-related ED visits was not significantly different among PLWH when compared with persons without HIV [aHR 1.22 (95% CI: 0.99–1.50), *P* = 0.07] (*Figure 3*).

The unadjusted rate (per 100 person-years) of all-cause death was higher among PLWH than among persons without HIV [10.3 (95% CI: 8.9–12.0) vs. 6.9 (95% CI: 6.5–7.3), respectively, *P* < 0.001]. This persisted in multivariable analysis, as PLWH were at significantly higher risk of all-cause death compared with persons without HIV [aHR 1.31 (95% CI: 1.08–1.58), *P* = 0.006] (*Figure 3*).

### Immunodeficiency severity and heart failure-related outcomes and death

When categorized based on CD4 cell count at the time of incident HF diagnosis in PLWH, the unadjusted rate (per 100 person-years) of HF hospitalizations was not materially different across baseline CD4 count, while the rate of HF-related ED visits was highest among those with <200 CD4 cells/μL (*Table 2*). An inverse relationship between baseline CD4 count and rate of all-cause death was also observed (*Table 2*).

**Table 2:** Unadjusted rates of clinical outcomes by baseline CD4 count in 448 persons living with HIV who developed incident heart failure

	Rate per 100 person-years (95% confidence interval)
Hospitalization for heart failure	
Baseline CD4 count, cells/μL	
<200	14.9 (11.1–19.9)
200–499	15.7 (13.0–19.0)
≥500	12.8 (10.2–16.2)
Missing	10.7 (6.2–18.4)
Emergency department visit related to heart failure	
Baseline CD4 count, cells/μL	
<200	48.2 (41.0–56.7)
200–499	30.1 (26.2–34.5)
≥500	32.6 (28.2–37.7)
Missing	20.6 (13.9–30.5)
All-cause death	
Baseline CD4 count, cells/μL	
<200	15.2 (11.4–20.3)
200–499	11.0 (8.8–13.8)
≥500	7.5 (5.5–10.1)
Missing	7.4 (3.9 –14.2)

In multivariable analyses, compared with persons without HIV, PLWH with CD4 count <200 cells/μL had significantly higher adjusted risks of all-cause death [aHR 1.83 (95% CI: 1.27–2.65), *P* = 0.001] and HF-related ED visits [aHR 1.62 (95% CI: 1.12–2.33), *P* = 0.01], but not HF hospitalization relative to persons without HIV (*Figure 3*). Persons living with HIV with CD4 count 200–499 cells/μL also had significantly higher adjusted risks of all-cause death [aHR 1.32 (95% CI: 1.01–1.72), *P* = 0.04], but not of HF hospitalization or HF-related ED visits. Persons living with HIV with baseline CD4 counts >500 cells/μL had no significant differences in adjusted risks of death, HF hospitalization, or HF-related ED visits when compared with persons without HIV (*Figure 3*).

## Discussion

Our contemporary, large, multicentre cohort study of incident HF provides important new insights about the potential influence of HIV infection on subsequent HF-related outcomes and death. We found that among adults with newly diagnosed HF, PLWH were diagnosed with HF at a younger age, were more likely to be female, and had a lower prevalence of clinical cardiovascular risk factors but a higher prevalence of major non-cardiac comorbidities than persons without HIV. While PLWH experienced a higher rate of all-cause death compared with persons without HIV, there were no significant differences in the adjusted risks of HF hospitalization or HF-related ED visits between the two groups.

While the incidence of developing HF appears to be higher among PLWH,^6^^,^^7^^,^^16^^,^^30^ to our knowledge, our study is the first to show that PLWH with HF in the contemporary ART era are at independently higher risk of death but not HF hospitalizations or HF-related ED visits when compared with the general incident HF population within the context of an integrated healthcare delivery framework. Our findings may be explained by multiple factors. Although the prevalence of traditional cardiovascular risk factors was higher among persons without HIV, the rates of non-cardiovascular conditions associated with excess mortality among persons with HF (including chronic liver disease, chronic lung disease, and cancer) were notably higher among PLWH.^31^ Persons living with HIV may have also accessed medical care more frequently due to the need for monitoring and management of their HIV disease when compared with persons without HIV, resulting in potentially more vigilant surveillance of HF-related symptoms and treatment of HF in ambulatory or ED settings precluding the need for hospitalization. The beneficial effects of overall coordinated care management of HF among those with or without HIV within the participating integrated healthcare delivery systems that are focused on prevention, early detection and evidence-based, guideline-driven care may have also contributed to the observed results demonstrating no difference in rates of HF hospitalization.

The evaluation of HF-related ED utilization separately from HF hospitalizations in PLWH with incident HF is novel. Emergency department visits are a unique endpoint associated with high acuity and shorter length-of-stay, representing both cause-specific and overall health care utilization. Previous investigations of Medicare beneficiaries suggest that a majority of ED presentations for HF result in subsequent hospitalization,^32^ but this is not generalizable to all populations, as rates of HF-related ED visits within our integrated healthcare delivery systems substantially outnumber HF hospitalizations.^33^ In this study, adults with incident HF—with or without HIV—experienced high rates of HF-related ED visits. While this may have reduced our specificity in establishing HF as the specific cause for excess ED utilization, by including this outcome we provide a more complete assessment of the overall burden of HF morbidity compared with previous studies. Our study supports the need to develop and validate accurate risk prediction models for both HF-related ED visits as well as HF hospitalization that are tailored to PLWH and could facilitate potential interventions aimed at reducing preventable emergency care, which is particularly relevant during the ongoing coronavirus disease 2019 pandemic.^34^

The excess risk of all-cause death observed among PLWH may have also been influenced by the effects of baseline immunodeficiency. After accounting for variation in baseline characteristics including sociodemographic features, comorbidity burden, and receipt of guideline-directed medical therapies for HF, there were no differences in adjusted risks of all-cause death, HF hospitalization, or HF-related ED visits among PLWH with baseline CD4 counts of ≥500 cells/μL when compared with persons without HIV. Our study extends previous single-centre studies and others that focused only on selected HF patients.^17^ The adjusted risks of death being higher among PLWH with low CD4 count (<500 cells/μL), supports the hypothesis that the excess risks of these outcomes were unlikely directly HF-related given that low CD4 count was not independently associated with a higher risk of HF hospitalizations or HF-related ED visits, and PLWH having low CD4 count are known to be at higher risk for premature death due to non-cardiovascular causes.^35^ In PLWH with severe immune deficiency, viruses may induce inflammatory damage, apoptosis, and mitochondrial dysfunction in myocardial cells.^12^^,^^36^^,^^37^ Persons living with HIV with low CD4 count may also experience higher rates of early left ventricular hypertrophy and diastolic dysfunction^38–40^—precursors to clinical HF—and CD4 count appears inversely related to LVEF and markers of chronic inflammation associated with myocardial fibrosis and HF.^41–44^ However, the actual clinical impact of these effects on HF prognosis among PLWH remains unclear, as we observed no differences in the risks of HF hospitalization or HF-related ED visits between PLWH and persons without HIV. Future studies are needed to identify potentially modifiable cardiac and non-cardiac factors related to overall survival and HF-related complications.

Our study had several limitations. While our cohort included insured adults receiving care in three large integrated healthcare delivery systems with combined membership of nearly 10 million, our results may not fully generalize to those who are uninsured or to other practice settings, particularly those outside of North America. However, the broad racial/ethnic and sociodemographic composition of our cohort is highly representative of the US HIV population and may be more generalizable than previous studies to typical contemporary community-based settings.^45^ We were also unable to assess the influence of changes in CD4 count or HIV RNA levels among PLWH after incident HF diagnosis. Time-varying immune status and time from diagnosis of HIV in relation to development of subsequent comorbid conditions may also influence outcomes among PLWH with incident HF and remains an area for future investigation. Information on LVEF was not available for approximately 40% of patients, precluding our ability to evaluate for an interaction between systolic function and the association between HIV status and HF-related morbidity and mortality. We also used a purposely broad definition of HF-related ED visits based on diagnostic codes to increase capture of potential HF complications. However, given that these encounters did not differentiate between the primary reason for the ED visit vs. a secondary reason or complicating comorbidity, it is possible that some ED visits were included that were not primarily for the purpose of worsening HF. Any misclassification bias, however, would have been non-differential and should not have materially affected our results. Finally, we cannot exclude the possibility of residual or unmeasured confounding.

In a large multicentre, contemporary US-based incident HF cohort involving patients receiving care within integrated healthcare delivery systems, PLWH experienced a higher adjusted rate of all-cause mortality—but not HF hospitalization or HF-related ED visits—compared with persons without HIV. These findings, however, may be explained, in part, by non-cardiovascular conditions associated with excess mortality among persons with HF that were noticeably higher among PLWH. Future research is needed to investigate the potential contribution of time-varying immune deficiency and varying types of ART on outcomes in PLWH, in addition to delineation of modifiable risk factors and accurate risk prediction models that can facilitate more personalized, optimized care for PLWH who develop HF.

## Lead author biography

**Figure:**
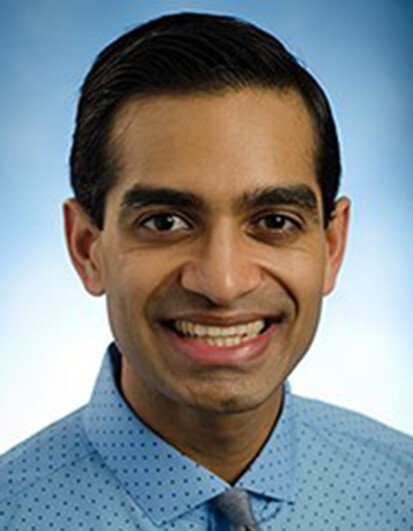


Dr Harshith R. Avula is an advanced heart failure and transplant cardiologist with The Permanente Medical Group of Northern California. He is an active collaborator with the Kaiser Permanente Division of Research, where his research has centred on the epidemiology and outcomes of select populations of adults with heart failure, and where he also serves as a co-investigator with ongoing clinical trials. As a public health scholar, Dr Avula remains focused on research identifying healthcare disparities and seeking to improve care and outcomes for vulnerable populations of adults with heart failure, including persons living with human immunodeficiency virus.
